# Lectures on Fevers

**Published:** 1878-01

**Authors:** 


*Lectures on Fevers.* By Alfred L. Loomis, A. M., M. D. New York: William Wood & Company. 1877. Buffalo: H. H. Otis. pp. 403.

"These lectures were delivered in the Medical Department of the University of the city of New York to the class of 1876-7." and were phonographically reported. They are an embodiment not only of the latest thought on the subjects of which they treat but we believe of the best thought and practice. The method and thoroughness of the author's work are apparent at a glance. The morbid anatomy of each fever is first taken up then its etiology, symptoms, differential diagnosis, prognosis and treatment. A Bibliography of fever literature published since 1850 finished the volume, in which, papers that have only appeared in medical journals are not included. The Bibliography comprises titles of works from between four and five hundred authors of eminence, and is in itself interesting and valuable, since there is no complete medical bibliography published.

"These lectures are the result of careful study of the literature referred to in the bibliography combined with extensive clinical experience."

This is without doubt the best treatise on fevers in the English language.

